# Intermolecular addition reactions of *N*-alkyl-*N*-chlorosulfonamides to unsaturated compounds

**DOI:** 10.3762/bjoc.11.136

**Published:** 2015-07-21

**Authors:** Gerold Heuger, Richard Göttlich

**Affiliations:** 1 Organisch-Chemisches Institut, WWU Münster, Corrensstraße 40, 48149 Münster, Germany; 2 Institut für Organische Chemie, Justus-Liebig-Universität Giessen, Heinrich-Buff-Ring 58, 35392 Giessen, Germany

**Keywords:** addition reactions, catalysis, *N*-chlorosulfonamides, haloamination, radical reaction

## Abstract

*N*-Alkyl-*N*-chlorosulfonamides add to alkenes under copper(I) catalysis. In reactions of styrene derivatives with terminal double bonds the addition products were obtained in excellent yield and high regioselectivity. Lower yields are obtained in addition reactions to non-aromatic alkenes. The reaction most likely proceeds via a redox catalysis and amidyl radicals, a concerted mechanism has been ruled out and a polar mechanism via chloronium ions would lead to the opposite regiochemistry.

## Introduction

In earlier publications we described the cyclisation of various unsaturated *N*-hetero-substituted amines and amides via radicals [1–3] and other mechanistic pathways [4–8]. Although reported by other groups [9–11] in our hands an efficient intermolecular addition reaction of *N*-hetero substituted amines via radicals was not possible in appreciable yields. The reactivity of the intermediate aminyl radicals towards alkenes was simply not high enough and various side reactions became predominant. We therefore turned our attention to more electrophilic, thus more reactive, nitrogen-centered radicals and chose sulfonamidyl radicals as such electrophilic intermediates for an efficient intermolecular addition reaction.

Addition reactions with *N*-alkyl-*N*-halosulfonamides to unsaturated compounds have not been examined in detail so far. In earlier works Komori added a secondary *N*-chloro-sulfonamide to 1-hexene under photoirridation [12–13] and Priestly [14], Seden [15] and Daniher [16] published addition reactions of secondary *N*-halosulfonamides and *N*,*N*-dihalosulfonamides to alkenes. Neale [17] discussed a radical mechanism via nitrogen radicals as intermediates. In a more recent series of publications Li developed a new aminohalogenation of cinnamic esters using *N*,*N*-dichloro-*p*-toluenesulfonamide and ZnCl_2_ or Cu(OTf)_2_ [18] as catalysts and transferred these conditions to reactions with alkynes [19–21] and α,β-unsaturated ketones [19–21]. An ionic mechanism via halonium ions was proposed. The amidofluorination of alkenes has been achieved by Zhang [22] using copper or palladium catalysts and proceeds via radicals and fluoropalladation, respectively. Cyclisation reactions of unsaturated sulfonamides which proceed via amidyl radicals have been described by Li [23] and by Oshima [24]. Chemler [25–26] discusses radical and polar pathways as competing mechanisms and has developed a nice copper-catalyzed oxidative amidation of alkenes whilst Muñiz [27] in a recent publication proposes a polar sulfonamido-chlorination mechanism of alkenes.

Similar, intramolecular and intermolecular additions of *N*-chlorosulfonamides and derivatives like Chloramine-T to alkenes have been described by Sharpless [28–29], Komatsu [30–32] and Dodd [33]. In these examples the nitrogen center carries no substituents, which limits the scope of the reactions. In our own studies we wanted to develop a radical addition, using amidyl radicals with an *N*-alkyl substituent, which we anticipated to generate readily from the corresponding *N*-chloroamides by electron transfer from copper(I) catalysts.

## Results and Discussion

**Scheme 1:**
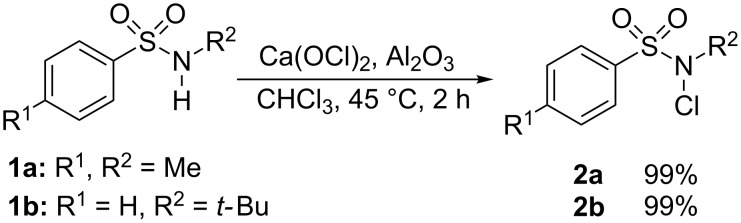
Preparation of the chloroamides.

The *N*-chlorosulfonamides can be easily prepared by reaction of the sulfonamide with calcium hypochlorite and moist alumina [34], which produced the corresponding *N*-chloro compounds **2a** and **2b** in quantitative yield (Scheme 1).

Other procedures for the synthesis of these compounds including *N*-chlorination with an fivefold excess of Oxone^®^ in the presence of NaCl/Al_2_O_3_ [35] or deprotonation and reaction with NCS [36–37] did not lead to higher or led in distinctly lower yields to the *N*-chloro compounds.

**Scheme 2:**
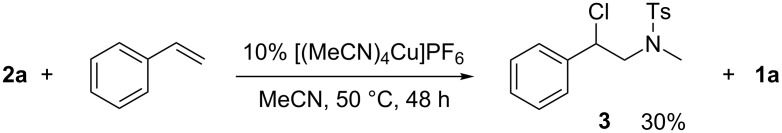
First experiments for the intermolecular radical addition.

We chose styrene as the model compound for the first addition reactions of *N*-chlorosulfonamide **2a** and used the complex [(MeCN)_4_Cu]PF_6_ as catalyst (Scheme 2). However even after a prolonged reaction time of 24 hours no addition product could be detected by TLC (Table 1, entry 1). Increasing the reaction temperature to 50 °C led to the formation of the addition product **3** in low yield. The yield was further raised to 30% by a longer reaction time of 48 h (Table 1, entries 2 and 3).

**Table 1:** Optimized addition reactions of **2a** with styrene.

Entry	**2a** (equiv)	Styrene (equiv)	Catalyst	Solvent	Reaction time (h)	Temperature (°C)	Yield of **3** (%)

1	1.2	1	10% [(MeCN)_4_Cu]PF_6_	MeCN	24	rt	–
2	1.2	1	10% [(MeCN)_4_Cu]PF_6_	MeCN	24	50	16
3	1.2	1	10% [(MeCN)_4_Cu]PF_6_	MeCN	48	50	30
4	1.2	1	50% [(MeCN)_4_Cu]PF_6_	MeCN	48	50	25
5	1	3	10% [(MeCN)_4_Cu]PF_6_	MeCN	48	75	43
6	1	3	10% CuCl	MeCN	48	75	60
**7**	**1**	**3**	**10% CuCl**	**PhCN**	**48**	**100**	**92**
8	1	1	10% CuCl	PhCN	48	100	48
9	2	1	10% CuCl	PhCN	48	100	34

Increasing the amount of catalyst led to no better results whilst rising the temperature to 75 °C and adding an excess of styrene increased the yield to 43%. Under these conditions the *N*-chlorosulfonamide **2a** was completely consumed, undesired products were the sulfonamide **1a** as well as oligostyrenes. The oligomerisation should be slowed down by using copper(I) chloride as the catalyst, which (after oxidation to copper(II) chloride) captures carbon radicals at a diffusion controlled rate [38].

Indeed using copper(I) chloride as the catalyst we obtained a yield of 60% of the addition product with sulfonamide **1a** being the remaining side product. We supposed that this sulfonamide was generated by H-abstraction from the solvent and therefore we used benzonitrile, a solvent from which hydrogen cannot easily be abstracted and which allowed us a higher reaction temperature (100 °C). Using these conditions we obtained a nearly quantitative yield of 92% of **3** (Table 1, entry 7), whilst changing the chloroamide/styrene ratio led to reduced yields.

**Scheme 3:**
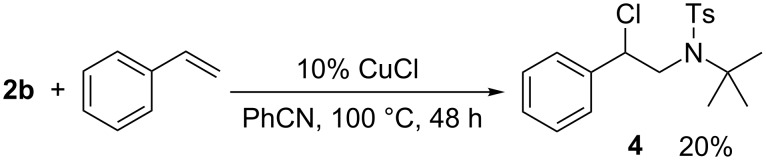
Reaction of sterically hindered *N*-chlorosulfonamides.

We next wanted to check the scope of this addition reaction and used, with the chloroamide **2b**, a sulfonamide with an extreme sterical hinderance (*tert*-butyl group). This led to the addition product **4** in a distinct lower yield (Scheme 3).

In a next step we added chloroamide **2a** to a variety of styrene derivatives (Table 2). Whilst the addition proceeds well with electron-poor styrenes such as 4-nitro and 4-fluorostyrene, an electron rich substrate with a methoxy substituent (Table 2, entry 4) leads to a complex mixture of products. Most likely the electron-rich aromatic ring is oxidized under these conditions, leading to a variety of products which could not be separated.

**Table 2:** Addition reactions of **2a** with styrene derivatives.

Entry	Olefine	Product	Yield (%)

1	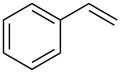	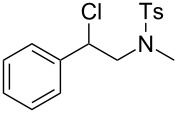 **3**	92
2	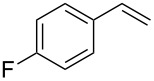	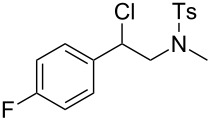 **5**	82
3	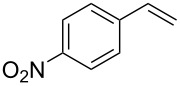	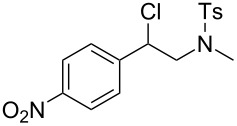 **6**	96
4	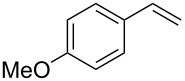	unidentified reaction mixture	–

With styrene derivatives giving high yields of addition products, we next turned our attention to the less reactive non-aromatic alkenes.

### Addition to non-aromatic alkenes

As expected non-aromatic alkenes are less good substrates for the radical addition of amidyl radicals and the yields of addition products of **2a** decreased significantly (Table 3). A conjugated diene like cyclooctadiene (Table 3, entry 4) and a terminal, sterically not hindered alkene like 1-decene (Table 3, entry 1) still gave reasonable yields of the addition product. The yield is lower in addition to norbornene and cyclooctene, both of which are more sterically hindered and do not allow any mesomeric stabilization of the intermediate radical (Table 3, entries 2 and 3). Addition to an alkyne (Table 3, entry 5) produced a complex mixture of products, which could not be separated. Electron poor alkenes like an unsaturated ketone (Table 3, entry 6) are not good substrates for the addition of amidyl radicals either, as amidyl radicals are expected to be electrophilic themselves. Therefore we expected the addition to electron-rich enol ethers to proceed smoothly, however, with such electron-rich alkenes polar reaction pathways seem to become predominant (Table 3, entries 8 and 9).

**Table 3:** Addition reactions of **2a** with electron-deficient olefines.

Entry	Olefine	Product^a^	Yield (%)

1	1-decene	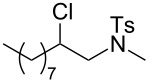 **7**	34
2	cyclooctene	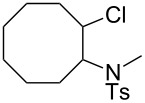 **8**	13
3	norbornene	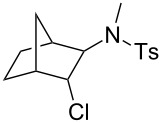 **9**	26 dr 2:1
4	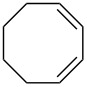	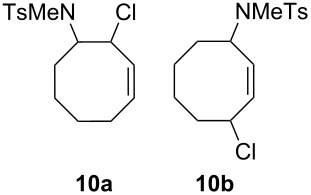	33 (**10a**) 10 (**10b**)
5	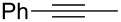	unidentified reaction mixture	–
6	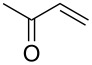	no addition	–
7	cyclohexene	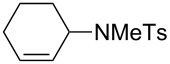 **11**	17
8	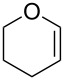	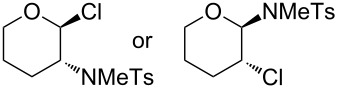 **12**	26
9	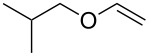	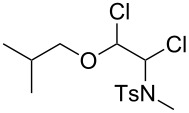 **13**	34

^a^All products are obtained as racemates.

**Scheme 4:**
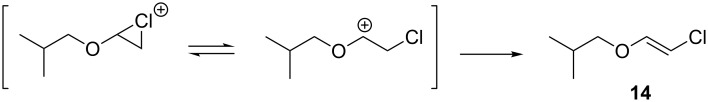
Proposed mechanism of the chlorination.

Whilst the formation of **11** from cyclohexene could result from an elimination of HCl after the radical addition due to the high reaction temperature, the formation of regioisomers of **12** and especially the formation of **13** can be explained by a halonium-ion transfer to the alkene. This could produce an chloro-substituted alkene **14**, which in a second step would undergo radical addition of **2b** (Scheme 4).

This result surprised us, as we did not regard the chloroamide as a chloronium ion source. For the addition reactions to styrenes a polar mechanism can be ruled out due to the observed regiochemistry, however, in all other cases a polar mechanism is possible too and we therefore wanted to verify the radical-type mechanism which we initially anticipated.

### Ionic or radical reaction mechanism

**Scheme 5:**
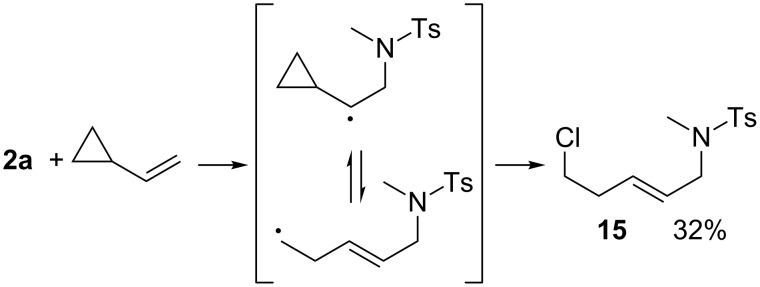
Ring opening in the case of cationic or radical intermediates.

For these studies we first chose vinylcyclopropane as the substrate to rule out a concerted mechanism, as this alkene upon addition of a radical or a cation should react fast under ring-opening [39], producing an acyclic product. Upon copper(I) catalyzed addition of **2a** to vinylcyclopropane only the ring-opend product **15** was obtained, which rules out a concerted mechanism and is, due to the observed regiochemistry, a strong indication for a radical pathway of the addition reaction (Scheme 5).

Another strong argument for a mechanism via radicals is the complete surpression of the reaction of **2a** with decene in the presence of TEMPO (2,2,6,6-tetramethyl-1-piperidinyloxy) as a radical trap, which we observed.

**Scheme 6:**
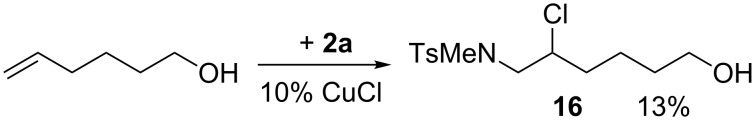
Addition to unsaturated alcohols prone to halocyclization.

In a last experiment we added **2a** to 5-hexen-1-ol, a substrate that easily reacts with halonium ions in a halocyclization [40] (Scheme 6).

We only obtained the simple addition product **16** with no traces of halocyclization, again ruling out a polar pathway.

## Conclusion

In summary we have shown that *N*-chlorosulfonamides can be added to styrenes efficiently under copper(I) catalysis via amidyl radicals. Addition to non-aromatic alkenes proceeds less readily in lower yield and electron-rich alkenes react via a competing polar reaction pathway.

## Experimental

All solvents were purified by distillation and dried, if necessary, prior to use. Products were purified by flash chromatography on silica gel (40–63 μm). ^1^H and ^13^C NMR spectra were recorded on Bruker WM 300 and ARX400 spectrometers or on a Varian Unity plus 600 spectrometer in CDCl_3_ using TMS as internal standard.

**Preparation of the *N*-chlorosulfonamides 2a, 2b; general procedure:** 5.72 g Ca(OCl)_2_ (40 mmol, 2 equiv), moist alumina (10 g) and chloroform (50 mL) were efficiently stirred at 40 °C for 10 min. The sulfonamide (20 mmol) was then added, and the mixture was stirred at 40 °C until the sulfonamide had disappeared (monitored by TLC (pentane/ether 1:1), 1.5–3 h). The crude product was separated from solids and purified by chromatography (silica gel, pentane/ether 3:1).

***N*-Chloro-*N*,4-dimethylbenzenesulfonamide (2a)** as white crystals. *R*_f_ 0.48 (pentane/ether 3:1); ^1^H NMR (400 MHz, CDCl_3_) δ 2.49 (s, 3H), 3.11 (s, 3H), 7.42 (d with fine-splitting, *J* = 8.0 Hz, 2H), 7.84 (d with fine-splitting, *J* = 8.4 Hz); ^13^C NMR (100 MHz, CDCl_3_) δ 21.6, 45.3, 128.5, 129.6, 129.8, 145.5.

***N*-*tert*-Butyl-*N*-chlorobenzenesulfonamide (2b)** as white crystals. *R*_f_ 0.52 (pentane/ether 3:1); ^1^H NMR (400 MHz, CDCl_3_) δ 1.50 (s, 9H), 7.52–7.57 (m, 2H), 7.62–7.66 (m, 1H), 7.99 (dd, *J* = 8.4, 1.2 Hz, 2H) ppm; ^13^C NMR (100 MHz, CDCl_3_) δ 29.1, 68.1, 128.80, 128.84, 133.5, 138.5.

**Metal-catalyzed addition reactions; general procedure:** In a heat-dried Schlenk vessel 0.1 equiv (related to the *N*-chlorosulfonamide) of the metal salt were dissolved in anhydrous benzonitrile under an Ar atmosphere and heated to 100 °C. To this solution the *N*-chlorosulfonamide and the olefine were added and stirred for 48 h at this temperature. The solvent was removed by bulb-to-bulb distillation and the residue was taken up in dichloromethane. Approx. 10 g of silica gel were added and the solvent was removed in vacuo. Purifying by flash chromatography (pentane/ether 10:1 to 1:1) gave the addition products as described below.

***N*-(2-Chloro-2-phenylethyl)-*N*-methylbenzenesulfonamide (3)** as a colourless oil [27]. *R*_f_ 0.47 (pentane/ether 3:1); ^1^H NMR (600 MHz, CDCl_3_) δ 2.41, (s, 3H), 2.62 (s, 3H), 3.40 (dd, *J* = 14.4, 7.2 Hz, 1H), 3.58 (dd, *J* = 14.4, 7.8, 1H), 5.10 (t, 7.2 Hz, 1H), 7.29–7.38 (m, 5H), 7.41 (m, 2H), 7.63 (d, *J* = 8.4 Hz, 2H); ^13^C NMR (150 MHz, CDCl_3_) δ 21.4, 36.8, 57.9, 61.2, 127.2, 127.4, 128.71, 128.78, 129.7, 134.6, 138.6, 143.5; MS (ESI, 1.57 kV, MeOH) *m*/*z* (%): 324 (38) [M^+^], 310 (20), 288 (70) [M − Cl^+^], 262 (8); HRMS (ESI) *m*/*z*: [M^+^] calcd for C_16_H_18_N^35^ClO_2_S, 324.0796; found, 324.0820. C_16_H_18_N^37^ClO_2_S [M^+^]: 326.0770; found: 326.0793. C_16_H_18_N^35^ClO_2_S + Na [M + Na]^+^, 346.0608; found, 346.0639; C_16_H_18_N^37^ClO_2_S + Na [M + Na]^+^, 348.0584; found, 348.0613; Anal. calcd for C_15_H_16_NClO_2_S (323.843): C, 59.34; H, 5.60; N, 4.33; found: C, 59.51; H, 5.69; N, 4.06.

***N*-*tert*-Butyl-*N*-(2-chloro-2-phenylethyl)benzenesulfonamide (4)** as a colourless oil. *R*_f_ 0.61 (pentane/ether 3:1); ^1^H NMR (400 MHz, CDCl_3_) δ 1.25 (s, 9H), 3.71 (dd, *J* = 15.6, 8.8 Hz, 1H), 3.91 (dd, *J* = 15.8, 4.6 Hz, 1H), 5.55 (dd, *J* = 8.8, 4.4 Hz, 1H), 7.32–7.41 (m, 5H), 7.46–7.56 (m, 3H), 7.93 (dd, *J* = 7.2, 1.2 Hz, 2H); ^13^C NMR (100 MHz, CDCl_3_) δ 29.5, 53.3, 59.7, 62.8, 127.1, 127.5, 128.62, 128.66, 128.9, 132.2, 139.2, 143.9; MS (ESI, 1.57 kV, MeOH) *m/z* (Intens. ×10^6^): 374 (1.15) [M + Na^+^], 338 (0.64) [M − Cl^+^], 316 (0.43), 282 (0.5), 260 (0.8); HRMS (ESI) *m*/*z*: [M + Na]^+^ calcd for C_18_H_22_N^35^ClO_2_S + Na, 374.0952; found, 374.0960; C_18_H_22_N^37^ClO_2_S + Na [M + Na]^+^, 376.0927; found, 376.0933.

***N*-[2-Chloro-2-(4-fluorphenyl)ethyl]-*N*-methylbenzenesulfonamide (5)** as a yellow oil. *R*_f_ 0.34 (pentane/ether 3:1); ^1^H NMR (300 MHz, CDCl_3_) δ 2.42 (s, 3H), 2.63 (s, 3H), 3.39 (dd, *J* = 14.5, 7.8 Hz, 1H), 3.55 (dd, *J* = 14.5, 6.9 Hz, 1H), 5.09 (t, *J* = 7.5 Hz, 1H), 7.05 (t, *J* = 8.5 Hz, 2H), 7.30 (d, *J* = 8.4 Hz, 2H), 7.39 (dd, *J* = 8.7, 5.1 Hz, 2H), 7.64 (d, *J* = 8.4 Hz, 2H); ^13^C NMR (75 MHz, CDCl_3_) δ 21.4, 37.0, 58.0, 60.4, 115.8 (d, *J* = 26.8 Hz), 127.3, 129.3 (d, *J* = 8.3 Hz), 129.8, 134.5, 134.7, 143.6, 162.8 (d, *J* = 246.8 Hz); ^19^F NMR (282 MHz, CDCl_3_) δ −112.79; MS (EI, 70 eV) *m/z* (%): 341 (3) [M^+^], 305 (7) [M − HCl^+^], 198 (97) [M − C_7_H_5_ClF^+^], 168 (2), 155 (71) [C_7_H_7_O_2_S^+^], 113 (19), 91 (100) [C_7_H_7_^+^], 65 (17) [C_5_H_5_^+^]; HRMS (ESI) *m/z*: [M + Na]^+^ calcd for C_16_H_17_N^35^ClFO_2_S + Na, 364.0545; found, 364.0574; C_16_H_17_N^37^ClFO_2_S + Na [M + Na]^+^, 366.0518; found, 366.0546; Anal. calcd for C_15_H_16_NClFO_2_S (341.834): C, 56.22; H, 5.01; N, 4.10; found, C, 56.43; H, 5.01; N, 4.05.

***N*-[2-Chloro-2-(3-nitrophenyl)ethyl]-*N*-methylbenzenesulfonamide (6)** as a yellow resin. *R*_f_ 0.14 (pentane/ether 3:1); ^1^H NMR (600 MHz, CDCl_3_) δ 2.43 (s, 3H), 2.69 (s, 3H), 3.50 (dd, *J* = 14.0, 8.0 Hz, 1H), 3.55 (dd, *J* = 14.4, 6.8 Hz, 1H), 5.21 (t, *J* = 7.6 Hz, 1H), 7.32 (d, *J* = 8.0 Hz, 2H), 7.59 (t, *J* = 8.0 Hz, 1H), 7.64 (d, *J* = 8.4 Hz, 2H), 7.80 (d, 7.6 Hz, 1H), 8.22 (m, 1H), 8.28 (m, 1H); ^13^C NMR (100 MHz, CDCl_3_) δ 21.4, 37.1, 57.8, 59.5, 122.5, 123.7, 127.3, 129.82, 129.88, 133.8, 134.3, 140.7, 143.9, 148.4; MS (EI, 70 eV) *m*/*z* (%): 368 (0.1) [M^+^], 198 (99) [M − C_7_H_5_ClNO_2_^+^], 170 (5) [C_7_H_5_ClNO_2_^+^], 155 (100) [C_7_H_7_O_2_S^+^], 127 (7), 91 (97) [C_7_H_7_^+^], 65 (16) [C_5_H_5_^+^]; Anal. calcd for C_16_H_17_N_2_ClO_4_S (368.841): C, 52.10; H, 4.65; N, 7.59; found, C, 52.35; H, 4.81; N, 7.40.

***N*-(2-Chlorodecyl)-4,*N*-dimethylbenzenesulfonamide (7)** as a colourless oil. *R*_f_ 0.60 (pentane/ether 3:1); ^1^H NMR (400 MHz, CDCl_3_) δ 0.86 (t, *J* = 6.9 Hz, 3H), 1.25–1.39 (m, 10H), 1.52–1.63 (m, 3H), 1.85–1.91 (m, 1H), 2.41 (s, 3H), 2.80 (s, 3H), 3.06 (dd, *J* = 14.1, 6.6 Hz, 1H) oder (10’), 3.34 (dd, *J* = 14.4, 6.6 Hz, 1H), 4.01–4.05 (m, 1H), 7.30 (d with fine-splitting, *J* = 7.8 Hz, 2H), 7.65 (d with fine-splitting, *J* = 8.4 Hz, 2H); ^13^C NMR (100 MHz, CDCl_3_) δ 14.0, 21.4, 22.6, 26.1, 29.0, 29.1, 29.3, 31.8, 35.3, 36.9, 56.8, 60.8, 127.3, 129.7, 134.4, 143.5; MS (EI, 70 eV) *m/z* (%): 359 (11) [M^+^], 310 (29) [M − CH_2_Cl^+^], 282 (3) [M − C_6_H_5_^+^], 246 (13), 198 (97), 186 (10), 155 (98) [C_7_H_7_O_2_S^+^], 91 (100) [C_7_H_7_^+^], 65 (38) [C_5_H_5_^+^]; HRMS (EI) *m*/*z*: [M^+^] calcd for C_18_H_30_N^35^ClO_2_S, 359.16778; found, 359.16858; Anal. calcd for C_18_H_30_NClO_2_S (359.961): C, 60.06; H, 8.40; N, 3.89; found, C, 60.29; H, 8.41; N, 3.90.

***N*-(2-Chlorocyclooctyl)-*N*-methylbenzenesulfonamide (8)** as a colourless oil. *R*_f_ 0.32 (pentane/ether 3:1); ^1^H NMR (600 MHz, CDCl_3_) δ 1.38–1.44 (m, 1H), 1.49–1.71 (m, 7H), 1.72–1.79 (m, 1H), 1.89–1.95 (m, 1H), 2.01–2.06 (m, 1H), 2.10–2.16 (m, 1H), 2.41 (s, 3H), 2.70 (s, 3H), 4.11 (m, 1H), 4.30 (m, 1H), 7.28 (d with fine-splitting, *J* = 8.4 Hz, 2H), 7.74 (d with fine-splitting, *J* = 8.4 Hz, 2H); ^13^C NMR (150 MHz, CDCl_3_) δ 21.4, 22.2, 24.7, 26.1, 27.8, 28.1 (broad), 30.85, 30.88, 62.2 (broad), 62.5, 127.4, 129.3, 137.1, 142.9; MS (EI, 70 eV) *m/z* (%): 329 (15) [M^+^], 294 (24) [M − Cl^+^], 272 (3) [M − C_4_H_9_^+^], 225 (8), 224 (47), 198 (5), 155 (21) [C_7_H_7_O_2_S^+^], 127 (42), 110 (42) [C_8_H_14_^+^], 84 (100), 57 (37) [C_4_H_9_^+^]; HRMS (EI) *m*/*z*: [M^+^] calcd for C_16_H_24_N^35^ClO_2_S, 329.12164; found 329.12040; Anal. calcd for C_16_H_24_NClO_2_S (329.891): C, 58.25; H, 7.33; N, 4.25; found: C, 58.47; H, 7.18; N, 4.03.

***N*-(3-Chlorobicyclo[2.2.1]hept-2-yl)-*N*-methylbenzenesulfonamide (9)** as a colourless oil. **9a** (endo-Product): *R*_f_ 0.26 (pentane/ether 3:1); ^1^H NMR (400 MHz, CDCl_3_) δ 1.29–1.37 (m, 2H), 1.40–1.60 (m, 3H), 1.86–1.93 (m, 2H), 2.39–2.41 (m, 1H), 2.42 (s, 3H), 2.78 (s, 3H), 3.71 (dd, *J* = 5.4, 1.8Hz, 1H), 4.06 (m, 1H), 7.30 (d with fine-splitting, *J* = 8.0 Hz, 2H), 7.71 (d with fine-splitting, *J* = 8.4 Hz, 2H); ^13^C NMR (100 MHz, CDCl_3_) δ 20.9, 21.4, 29.3, 30.5, 35.8, 39.7, 43.1, 64.9, 69.1, 127.3, 129.5, 135.9, 143.2; MS (EI, 70 eV) *m/z* (%): 313 (36) [M^+^], 278 (6) [M − Cl^+^], 250 (9) [M − C_2_H_4_Cl^+^], 238 (21), 224 (5), 198 (51), 158 (100) [M − C_7_H_7_O_2_S^+^], 131 (13), 121 (31), 91 (88) [C_7_H_7_^+^], 65 (20) [C_5_H_5_^+^]; HRMS (EI) *m*/*z*: [M^+^] calcd for C_15_H_20_N^35^ClO_2_S, 313.09033; found, 313.08982.

**9b** (exo-Product): *R*_f_ 0.42 (pentane/ether 3:1); ^1^H NMR (400 MHz, CDCl_3_) δ 1.19–1.22 (m, 3H), 1.44–1.54 (m, 1H), 1.59–1.72 (m, 1H), 1.93 (m, 1H), 2.06 (m, 1H), 2.42–2.44 (m, 1H), 2.42 (s, 3H), 2.93 (s, 3H), 4.06 (m, 1H), 4.14 (m, 1H), 7.29 (d with fine-splitting, *J* = 8.0 Hz, 2H), 7.86, d with fine-splitting, *J* = 8.0 Hz, 2H); ^13^C NMR (100 MHz, CDCl_3_) δ 21.4, 25.6, 29.2, 31.7, 34.7, 38.6, 45.0, 63.2, 68.5, 127.0, 129.5, 136.8, 143.0; MS (EI, 70 eV) *m*/*z* (%): 313 (50) [M^+^]; 278 (10) [M − Cl^+^]; 250 (6) [M − C_2_H_4_Cl^+^], 238 (31), 224 (7), 198 (67), 158 (100) [M − C_7_H_7_O_2_S^+^], 139 (11), 122 (34), 91 (88) [C_7_H_7_^+^], 65 (15) [C_5_H_5_^+^].

***N*-(4-Chlorocyclooct-2-enyl)-*N*-methylbenzenesulfonamide (10b)** as a colourless oil. *R*_f_ 0.32 (pentane/ether 3:1); ^1^H NMR (600 MHz, CDCl_3_) δ 1.49–1.60 (m, 4H), 1.63–1.71 (m, 2H), 1.73–1.79 (m, 1H), 2.12–2.17 (m, 1H), 2.40 (s, 3H), 2.74 (s, 3H), 4.72–4.80 (m, 2H), 5.19 (ddd, *J* = 11.1, 8.4, 1.2 Hz, 1H), 5.50 (ddd, *J* = 10.8, 7.8, 1.2 Hz, 1H), 7.27 (d, *J* = 8.4 Hz, 2H), 7.63 (d, *J* = 8.4 Hz); ^13^C NMR (150 MHz, CDCl_3_) δ 21.4, 23.6, 25.0, 28.8, 34.4, 40.1, 54.5, 56.7, 125.2, 127.3, 129.5, 132.8, 135.7, 143.2; MS (EI, 70 eV) *m*/*z* (%): 327 (36) [M^+^], 292 (14) [M − Cl^+^], 250 (26) [M − C_6_H_5_^+^], 231 (4), 224 (12) [M − C_8_H_7_^+^], 198 (8), 172 (43) [M − C_7_H_7_O_2_S^+^], 127 (45), 106 (16), 91 (100) [C_7_H_7_^+^], 57 (37) [C_4_H_9_^+^]; HRMS (Schubstange) *m/z* [M^+^]: calcd for C_16_H_22_N^35^ClO_2_S, 327.10599; found, 327.10532.

**Byproduct:* N*-(2-Chlorocyclooct-3-enyl)-*N*-methylbenzenesulfonamide (10a)** as a colourless oil. *R*_f_ 0.39 (pentan/ether 3:1); ^1^H NMR (300 MHz, CDCl_3_) δ 1.26–1.35 (m, 1H), 1.57–1.76 and 1.85–1.92 (m, 6H), 2.21–2.28 (m, 1H), 2.42 (s, 3H), 2.76 (s, 3H), 4.44–4.51 (m, 1H), 4.90–5.03 (m, 1H), 5.66 (ddd, *J* = 12.3, 6.5, 1.6 Hz, 1H), 5.77–5.86 (m, 1H), 7.29 (d, *J* = 8.1 Hz, 2H), 7.73 (d, *J* = 7.8 Hz, 2H); ^13^C NMR (150 MHz, CDCl_3_) δ 21.9, 23.5, 29.1, 30.3, 37.5, 57.3, 62.1, 127.2, 128.3, 129.4, 131.8 (selected peaks); MS (ESI, 1.30 kV, MeOH) *m/z* (Intens. ×10^6^): 677 (2.2) [2*M + Na^+^], 350 (3.1) [M + Na^+^], 314 (1.8) [MNa − Cl^+^].

***N*-Cyclohex-2-enyl-*N*-methylbenzenesulfonamide (11)** as a colourless oil [41]. *R*_f_ 0.42 (pentane/ether 3:1); ^1^H NMR (600 MHz, CDCl_3_) δ 1.46–1.52 (m, 1H), 1.57–1.61 (m, 1H), 1.74 (m, 2H), 1.93 (m, 2H), 2.43 (s, 3H), 2.70 (s, 3H), 5.11 (m, 1H), 5.57 (m, 1H), 5.81 (m, 1H), 7.30 (d, *J* = 8.4 Hz, 2H), 7.71 (d with fine-splitting, *J* = 8.4 Hz, 2H); ^13^C NMR (150 MHz, CDCl_3_) δ 21.3, 21.4, 24.3, 26.7, 29.1, 54.2, 127.0, 127.1, 129.6, 132.3, 137.2, 142.9; MS (EI, 70 eV) *m*/*z* (%): 265 (15) [M^+^], 237 (100) [M − C_2_H_4_^+^], 213 (24) [M − C_4_H_4_^+^], 186 (6), 155 (27) [C_7_H_7_O_2_S^+^], 126 (25) [M − C_7_H_7_OS^+^], 110 (66) [C_7_H_12_N^+^], 91 (75) [C_7_H_7_^+^], 55 (25); HRMS (ESI) *m*/*z*: [M + Na^+^] calcd for C_14_H_19_NO_2_SNa, 288.1029; found, 288.0984.

***N*-(2-Chlorotetrahydropyran-3-yl)-*N*-methylbenzenesulfonamide (12)** as a yellow oil. *R*_f_ 0.21 (pentane/ether 3:1); ^1^H NMR (400 MHz, CDCl_3_) δ 1.63–1.73 (m, 2H), 1.87–1.97 (m, 1H), 2.42 (s, 3H), 2.42–2.46 (m, 1H), 2.75 (s, 3H), 3.56–3.62 (m, 1H), 3.77 (ddd, *J* = 11.6, 11.6, 4.6 Hz, 1H), 3.90–3.96 (m, 1H), 5.10 (d, *J* = 9.2 Hz, 1H), 7.29 (d, *J* = 8.0 Hz, 2H), 7.77 (d with fine-splitting, *J* = 8.4 Hz, 2H); ^13^C NMR (100 MHz, CDCl_3_) δ 21.4, 26.4, 28.0, 34.6, 54.3, 67.5, 89.0, 127.8, 129.3, 136.2, 143.3; MS (EI, 70 eV) *m*/*z* (%): 303 (80) [M^+^], 259 (4) [M − C_2_H_4_O^+^], 241 (11), 214 (14), 196 (12), 155 (31) [C_7_H_7_O_2_S^+^], 127 (41), 108 (100), 91 (66) [C_7_H_7_^+^], 55 (42) [C_3_H_3_O^+^]; HRMS (EI) *m*/*z*: [M^+^] calcd for C_13_H_18_N^35^ClO_3_S, 303.06958; found, 303.06931.

***N*-(1,2-Dichloro-2-isobutoxyethyl)-*N*-methylbenzenesulfonamide (13)** as a yellow oil. *R*_f_ 0.56 (pentane/ether, 3:1); ^1^H NMR (400 MHz, CDCl_3_) δ 0.88 (d, *J* = 1.6 Hz, 3H), 0.89 (d, *J* = 1.6 Hz, 3H), 1.85 (sept, *J* = 6.6 Hz, 1H), 2.44 (s, 3H), 2.76 (s, 3H), 3.20 (d, *J* = 6.8 Hz, 2H), 5.30 (d, *J* = 6.4 Hz, 1H), 5.58 (d, *J* = 6.4 Hz, 2H), 7.32 (d, *J* = 8.4 Hz, 2H), 7.77 (d with fine-splitting, *J* = 8.4 Hz, 2H); ^13^C NMR (100 MHz, CDCl_3_) δ 19.0, 19.1, 21.4, 27.6, 28.0, 71.2, 76.5, 89.6, 127.5, 129.5, 136.1, 143.8; MS (ESI, 1.30 kV, MeOH) *m/z* (%): 376 (60) [M + Na^+^], 318 (11), 304 (3) [M − C_4_H_8_O^+^], 285 (6) [M − C_7_H_7_^+^], 262 (6), 226 (60), 208 (4), 197 (11), 187 (4), 163 (9), 155 (56) [C_7_H_7_O_2_S^+^], 139 (11) [C_7_H_7_OS^+^], 128 (4), 106 (9), 91 (6) [C_7_H_7_^+^], 72 (58) [C_4_H_8_O^+^], 57 (17) [C_4_H_9_^+^]; HRMS (ESI) *m*/*z*: [M + Na]^+^ calcd for C_14_H_21_N^35^Cl_2_O_3_S + Na, 376.0511; found, 376.0506; C_14_H_21_N^37^Cl_2_O_3_S + Na [M + Na]^+^, 378.0483; found, 378.0480.

***N*-(5-Chloropent-2-enyl)-*N*-methylbenzenesulfonamide (15)** as a yellow liquid and a non separable 4:1 mixture of isomers. *R*_f_ 0.23 (pentane/ether 3:1). Main isomer **15a** (probably the *E*-isomer): ^1^H NMR (400 MHz, CDCl_3_) δ 2.43 (s, 3H), 2.45–2.51 (m, 2H), 2.66 (s, 3H), 3.50 (t, *J* = 6.6 Hz, 2H), 3.60 (d, *J* = 6.4 Hz, 2H), 5.43–5.53 (m, 1H), 5.56–5.65 (m, 1H), 7.32 (d, *J* = 8.0 Hz, 2H), 7.67 (d with fine-splitting, *J* = 8.4 Hz, 2H); ^13^C NMR (100 MHz, CDCl_3_) δ 21.4, 34.1, 35.1, 43.7, 52.0, 127.45, 127.48, 129.61, 131.0, 134.5, 143.3. Minor isomer **15b** (probably the *Z*-isomer)(selected Peaks): ^1^H NMR (400 MHz, CDCl_3_) δ 2.44 (s, 3H), 2.67 (s, 3H), 3.51 (t, *J* = 6.6 Hz, 2H), 3.68 (d, *J* = 6.4 Hz, 2H), 7.33 (d, *J* = 8.0 Hz, 2H), 7.67 (d, *J* = 8.0 Hz, 2H); ^13^C NMR (100 MHz, CDCl_3_) δ 30.3, 34.2, 46.8, 126.7, 129.68, 130.2, 143.4; MS (EI, 70 eV) *m*/*z* (%): 287 (2) [M^+^], 272 (5) [M − CH_3_^+^], 238 (8) [M − CH_2_Cl^+^], 224 (16) [M − C_2_H_4_Cl^+^], 198 (14), 186 (100), 155 (58) [C_7_H_7_O_2_S^+^], 132 (53), 91 (93) [C_7_H_7_^+^], 57 (46) [C_4_H_9_^+^]; HRMS (ESI) *m*/*z*: [M + Na]^+^ calcd for C_13_H_18_N^35^ClO_2_S + Na, 310.0639; found, 310.0620; C_13_H_18_N^37^ClO_2_S + Na [M + Na]^+^, 312.0611; found, 312.0594; Anal. calcd for C_13_H_18_NClO_2_S (287.806): C, 54.25; H, 6.30; N, 4.87; found, C, 54.18; H, 6.40; N, 5.02.

***N*-(2-Chloro-6-hydroxyhexyl)-*N*-methylbenzenesulfonamide (16)** as a colourless oil. *R*_f_ 0,06 (pentane/ether 3:1); ^1^H NMR (400 MHz, CDCl_3_) δ 1.49–1.79 (m, 6H), 1.92–2.01 (m, 1H), 2.44 (s, 3H), 2.83 (s, 3H), 3.08 (dd, *J* = 14.0, 6.4 Hz, 1H), 3.39 (dd, *J* = 14.2, 7.0 Hz, 1H), 3.67 (t, *J* = 6.2 Hz, 2H), 4.05–4.11 (m, 1H), 7.33 (d, *J* = 8.0 Hz, 2H), 7.67 (d with fine-splitting, *J* = 8.8 Hz, 2H); ^13^C NMR (100 MHz, CDCl_3_) δ 21.4, 22.2, 32.0, 34.8, 36.9, 56.6, 60.4, 62.4, 127.3, 129.7, 134.4, 143.6; MS (EI, 70 eV) *m*/*z* (%): 318 (1) [M^+^], 283 (16) [M − Cl^+^], 270 (12), 246 (3), 198 (88), 186 (8), 155 (100) [C_7_H_7_O_2_S^+^], 127 (15), 91 (93) [C_7_H_7_^+^], 65 (43) [C_5_H_5_^+^]; HRMS (ESI) *m*/*z*: [M + Na]^+^ calcd for C_14_H_22_N^35^ClO_3_S + Na 342.0901; found, 342.0890; C_14_H_22_N^37^ClO_3_S + Na [M + Na]^+^: 344.0874; found, 344.0864.
